# Electron Tomography of Fusiform Vesicles and Their Organization in Urothelial Cells

**DOI:** 10.1371/journal.pone.0032935

**Published:** 2012-03-12

**Authors:** Samo Hudoklin, Kristijan Jezernik, Josef Neumüller, Margit Pavelka, Rok Romih

**Affiliations:** 1 Faculty of Medicine, Institute of Cell Biology, University of Ljubljana, Ljubljana, Slovenia; 2 Department of Cell Biology and Ultrastructure Research, Center for Anatomy and Cell Biology, Medical University of Vienna, Vienna, Austria; Institute of Molecular and Cell Biology, Singapore

## Abstract

The formation of fusiform vesicles (FVs) is one of the most distinctive features in the urothelium of the urinary bladder. FVs represent compartments for intracellular transport of urothelial plaques, which modulate the surface area of the superficial urothelial (umbrella) cells during the distension-contraction cycle. We have analysed the three-dimensional (3D) structure of FVs and their organization in umbrella cells of mouse urinary bladders. Compared to chemical fixation, high pressure freezing gave a new insight into the ultrastructure of urothelial cells. Electron tomography on serial sections revealed that mature FVs had a shape of flattened discs, with a diameter of up to 1.2 µm. The lumen between the two opposing asymmetrically thickened membranes was very narrow, ranging from 5 nm to 10 nm. Freeze-fracturing and immunolabelling confirmed that FVs contain two opposing urothelial plaques connected by a hinge region that made an omega shaped curvature. In the central cytoplasm, 4–15 FVs were often organized into stacks. In the subapical cytoplasm, FVs were mainly organized as individual vesicles. Distension-contraction cycles did not affect the shape of mature FVs; however, their orientation changed from parallel in distended to perpendicular in contracted bladder with respect to the apical plasma membrane. In the intermediate cells, shorter and more dilated immature FVs were present. The salient outcome from this research is the first comprehensive, high resolution 3D view of the ultrastructure of FVs and how they are organized differently depending on their location in the cytoplasm of umbrella cells. The shape of mature FVs and their organization into tightly packed stacks makes them a perfect storage compartment, which transports large amounts of urothelial plaques while occupying a small volume of umbrella cell cytoplasm.

## Introduction

Superficial urothelial cells (umbrella cells) of the urinary bladder contain numerous fusiform vesicles (FVs), called also fusiform vacuoles or discoidal vesicles [1], [2], [3]. FVs have been described, depending on mammalian species, as being either fusiform or discoidal in cross-section [4]. According to Staehelin et al., they have a form of biconvex discs with a diameter 0.5–1 µm [5], [6]. Minsky and Chlapowsky proposed that FVs are pancake-like flattened spheres, but this has never been confirmed by ultrastructural 3D analyses [7].

FVs are lined by an asymmetric unit membrane (AUM), which contains four major integral proteins, uroplakins (UPs) Ia, Ib, II and IIIa [8], [9], [10], [11], [12], [13]. Uroplakins form 16-nm intramembranous uroplakin particles, which are hexagonally arranged in urothelial plaques. Plaques measure between 0.3 and 1 µm in diameter [5], and they are connected by a non-thickened membrane, called hinge region [1], [14], [15]. UPs are synthesized in the endoplasmic reticulum, where UPIa and UPIb form heterodimers with UPII and UPIIIa, respectively. Conformational changes in the Golgi apparatus enable the formation of 16-nm intramembranous particles [1], [6], [15], [16], [17], which are hexagonally arranged into 2D crystalline plaques in the post-Golgi compartments [18]. While the structure of the 16-nm particles is largely known [19], the information on the 3D structure of mature FVs is missing.

The plaque composition of mature FVs is identical to that of the apical plasma membrane of umbrella cells, therefore it has been proposed that FVs are transported from the Golgi apparatus towards the apical cell surface where they fuse with the plasma membrane [1], [2], [15], [20], [21], [22]. According to one hypothesis, FVs are inserted into the apical plasma membrane during bladder distension (filling with urine) and retrieved during bladder contraction (micturition). This membrane recycling therefore provides a mechanism to adjust surface area of umbrella cells during distension-contraction cycles of the urinary bladder [1], [7], [15], [23], [24]. Alternative hypothesis says that FVs are not retrieved during contraction of the bladder; instead the apical surface area is accommodated only by the apical plasma membrane infolding [2]. The analyses of morpho-functional organization of FVs are therefore essential for understanding their role in the intracellular membrane traffic and in the turn-over of the apical plasma membrane.

Electron tomography (ET), which allows 3D reconstructions of objects with the resolution below 10 nm, has greatly contributed to the understanding of subcellular structures and compartments [25], [26], [27], [28]. In order to analyse subcellular structures by ET in the state ‘close to native’, samples should be fixed by high pressure freezing, which allows immobilization within milliseconds, followed by freeze substitution [29]. Because FVs are relatively large compartments, their 3D reconstruction requires serial sectioning and joining of tomograms.

Here we demonstrate that high pressure freezing and ET of serial sections, supported by freeze-fracture and immunocytochemistry, give a new insight into the structure and organization of FVs in umbrella cells. We also compared the arrangement of FVs during distension-contraction cycle of the urinary bladder, and analysed intermediate cells with respect to the occurrence of FVs.

## Results

### High pressure freezing gives a new insight into the ultrastructure of urothelial cells

**Figure 1:**
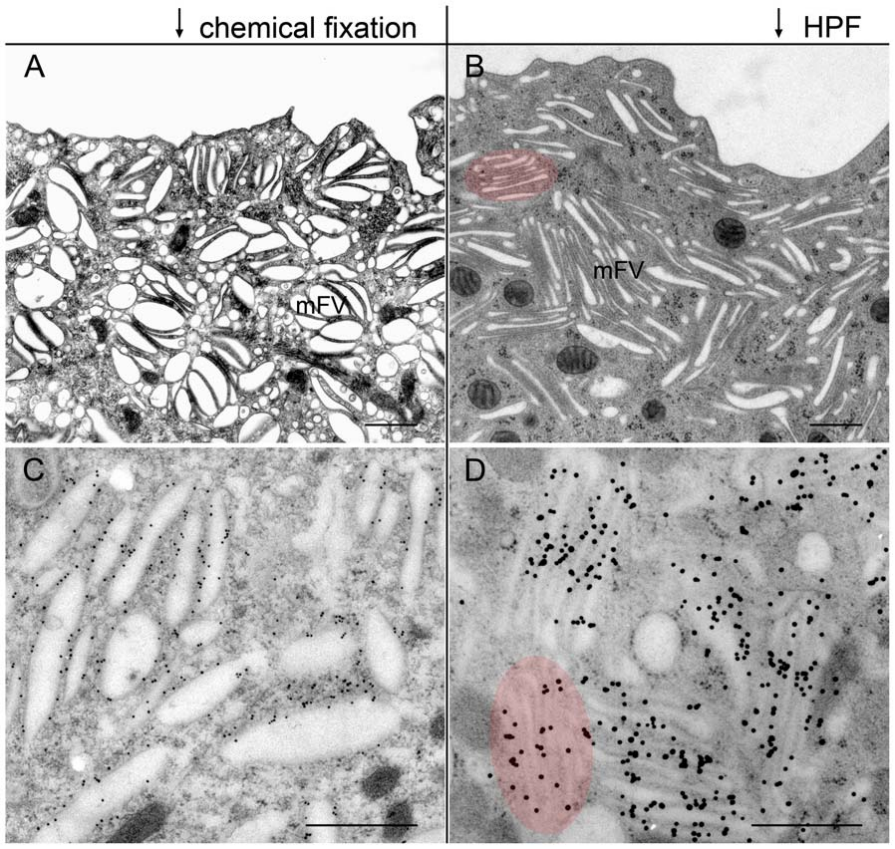
Comparison of chemically and HPF fixed umbrella cells of the urothelium. mFVs are the most prevailing compartments in chemically (A, C) and in HPF fixed umbrella cells (B, D). Regarding the ultrastructure, mFVs are more dilated in chemically fixed (A, C) than in HPF fixed samples (B, D). Regarding immunolabelling with anti-AUM antibody, pattern and density of labelling is comparable on chemically (C) and HPF fixed samples (D). The organization of mFVs into stacks is better preserved in HPF fixed samples (red over-colour in B and D). Bars: 500 nm.

**Figure 2:**
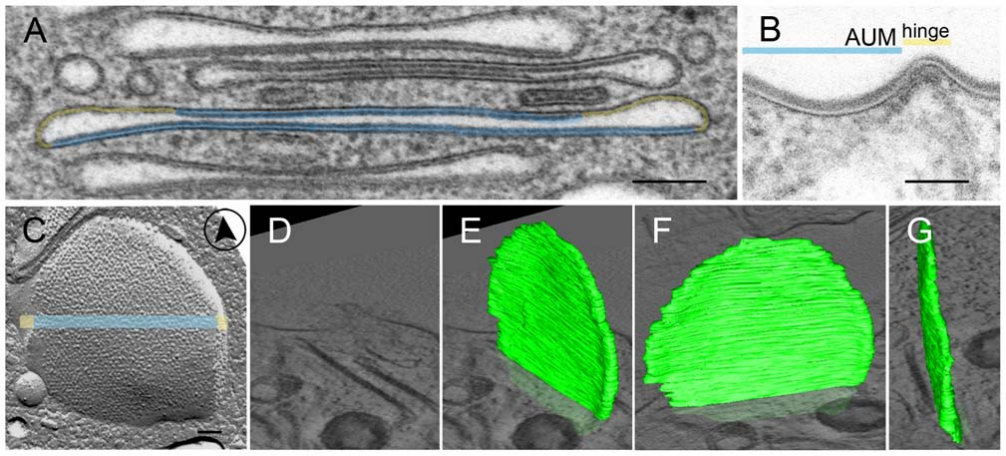
Structure of a mFV. (A) mFVs are flattened vesicles with two opposing plaques of thickened asymmetric unit membrane (AUM, blue) and slightly dilated, omega shaped hinge region of un-thickened membrane (yellow). (B) AUM and hinge regions can be seen also in the apical plasma membrane. (C) By freeze-fracturing, uroplakin particles are seen concentrated in the centre of the mFV (blue), while thin hinge region contains particle-free membranes (yellow). (D–G) Three-dimensional model of mFV shows that it has the shape of a flattened disk. In D, a slice from a tomogram is shown. In E–G, a 3D model of a mFV in different projections is shown (green). Bars: 100 nm in A, C; 50 nm in B.

Rapid immobilization of structures by cryo-fixation is currently the method of choice for EM studies of biological tissues. In this work, we compared the ultrastructure of urothelial cells obtained by high pressure freezing (HPF) followed by freeze substitution (FS) with that obtained by chemical fixation followed by ethanol dehydration. In both cases, the specimens were embedded in Epon. The general appearance of the urothelium was comparable; a clear distinction between the structure of basal, intermediate and umbrella cells was seen. On the other hand, samples prepared by the two fixation methods differed significantly in details (Figs. 1A, B). In samples prepared by HPF-FS, numerous vesicles were flat, with thickened and non-thickened membrane domains clearly discriminated in cross-sections (Figs. 1B, 2A, B). In chemically fixed cells, vesicles were often dilated (Fig. 1A). The ultrastructure of other membrane compartments, such as Golgi apparatus, endoplasmic reticulum, mitochondria, were also superior after HPF-FS compared to chemical fixation.

Next, we compared HPF-FS and chemical fixation, followed by Lowicryl embedding, for their use in immunocytochemistry. Urothelial cells immunolabelled with anti-AUM antibody in both cases occupied the same location and yielded comparable densities of gold particles (Figs. 1C, D). The difference between HPF-FS and chemically fixed, Lowicryl embedded samples was not in the density of the immunolabelling, but in the preservation of the ultrastructure. In the HPF-FS samples, anti-AUM labelled vesicles were flattened, while in the chemically fixed samples, vesicles were more dilated (Figs. 1C, D).

### Mature fusiform vesicles of umbrella cells are flattened discs

Umbrella cells contained numerous mature FVs (mFVs), recognized as elongated structures measuring up to 1.2 µm in their transverse diameter (Fig. 2A). Their lumen appeared as a very narrow slit (ranging 5–10 nm in the central region of the vesicle), slightly dilated only at the rims of vesicles (Fig. 2A). The two opposing membranes were clearly seen as 12 nm thick AUM, identical to those seen on the apical cell surface (Figs. 2A, B). Immunolabelling showed that flattened parts of mFVs were densely labelled with anti-AUM antibodies (Figs. 1C, D). Furthermore, freeze-fracturing showed that 16-nm uroplakin particles formed the central region of mFVs (Fig. 2C). On the other hand, the rounded, omega shaped rim of mFVs, contained non-thickened membrane, which was UP-negative and smooth on freeze-fracture replicas (Figs. 2A, C, 5A).

On HPF-FS urothelial samples, we performed ET to study the 3D structure of mFVs. Since FVs measured up to 1.2 µm in diameter, we had to join 3 to 5 serial tomograms, each 300 nm thick, in order to reconstruct the whole volume of an mFV (Table S1). In the model of an individual mFV, it was seen that it had the shape of a flattened disc, which is circular when viewed from the side (Figs. 2D–G, Movies S1, S2). The rim of the vesicle was slightly dilated.

### The organization of mature fusiform vesicles changes between central and subapical cytoplasm of umbrella cells

**Figure 3:**
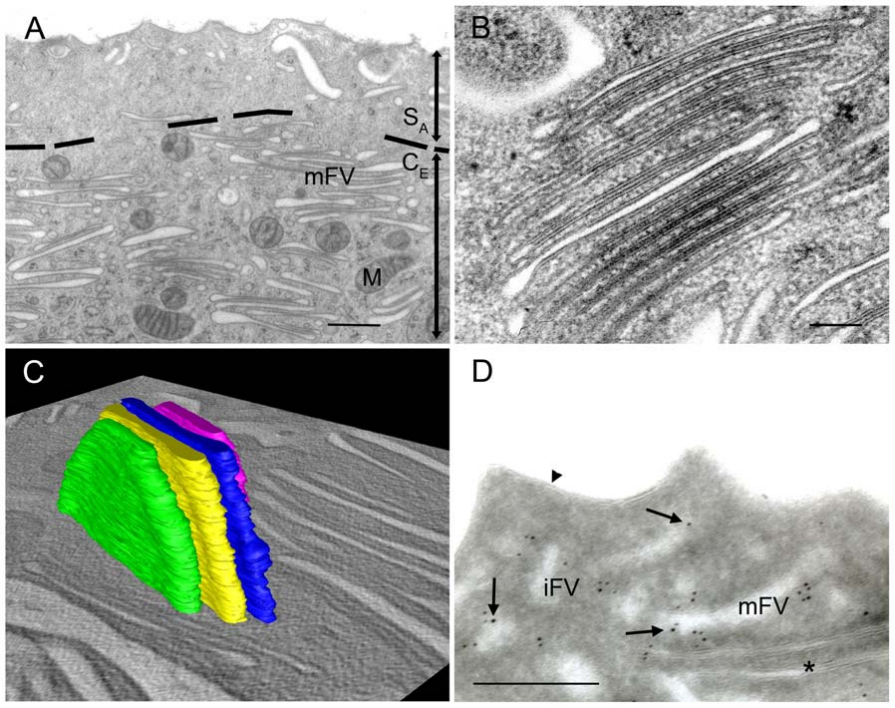
Stacks of mFV in the central cytoplasm of umbrella cells. (A) Cytoplasm of umbrella cells is divided into two regions: sub-apical (S_A_) and central (C_E_). Central cytoplasm contains majority of mFVs and other organelles. The border between the sub-apical and central cytoplasm is shown by a dashed line. (B) In the central cytoplasm, mFVs are often arranged into stacks. (C) Three-dimensional model of a stack shows that mFVs grouped into a stack have the same shape of flattened disk as individually positioned mFVs. Model of four stacked mFVs is presented. (D) Immunolabelling with anti-Rab27b antibody shows positive reactions (arrows) on some mFVs and some iFVs, while there is no labelling seen on the apical plasma membrane (arrowhead). Legend: M – mitochondrion, asterisk – mFV without anti-Rab27b labelling. Bars: 250 nm.

Mature FVs in umbrella cells had the morphology of flattened disks. However, there were two distinct regions of the cytoplasm regarding the organization of mFVs: central and subapical (Fig. 3A). The border between central and subapical cytoplasm corresponded to the position of the cytokeratin 20 network [30], [31], which was seen as a narrow line by anti-CK20 immunofluorescence (Fig. S1).

In the central cytoplasm of umbrella cells, mFVs were usually organized into stacks, where mFVs were closely packed in parallel arrangement to each other (Fig. 3B). Stacks were more obvious in the HPF-FS than in chemically fixed umbrella cells (Figs. 1B, D versus Figs. 1A, C). Stacks contained 4 to 15 FVs of similar lengths. mFVs in stacks were densely labelled with anti-AUM antibody (Fig. 1D). Stacks of mFVs were not surrounded by other membrane compartments, which suggested that they represented a relatively static, mature situation. Three-dimensional reconstructions showed that mFVs in stacks have the same flattened-disk shape as individually positioned mFVs (Fig. 3C, Table S1and Movies S3, S4).

In the subapical cytoplasm of umbrella cells, mFVs were sparser than in the central cytoplasm, and were usually seen as individual entities (Fig. 3A).

Immunolabelling of ultrathin cryo-sections with anti-Rab27b antibody showed that some mFVs from both central and subapical region were labelled (Fig. 3D), while some mFVs remained non-labelled. In addition, weak anti-Rab27b labelling was observed also on some immature FVs.

### Orientation of mature fusiform vesicles changes during distension-contraction cycle of the urinary bladder

**Figure 4:**
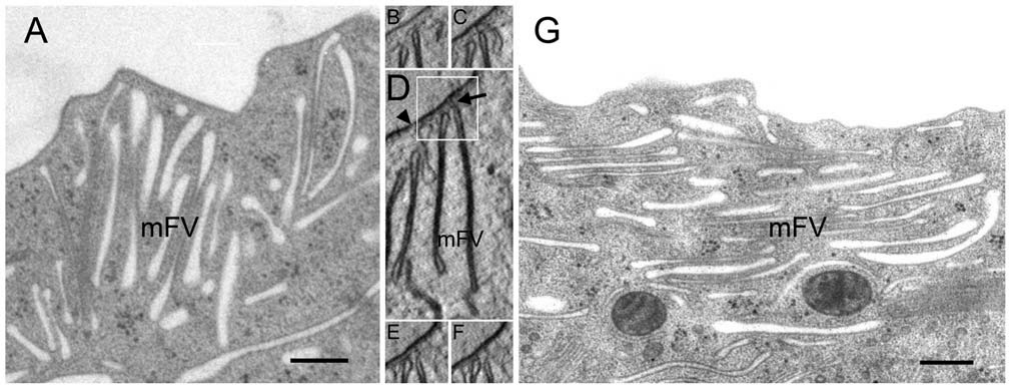
Orientation of mFVs in the sub-apical cytoplasm changes during bladder stretching. In umbrella cells of the contracted bladders (A–F), mFVs are oriented mostly perpendicular to the apical plasma membrane, while in the distended bladders (G), mFV are oriented mostly parallel to the apical plasma membrane. Panels B–F show representative slices from the tomogram. In panel D, a contact (arrow) between mFVs and the apical plasma membrane (arrowhead) is seen. White box in D corresponds to the area shown in B, C, E and F. Bars: 250 nm.

We compared the organization of mFVs in the umbrella cells of distended and contracted bladders. In contrast to other studies, which employed different solutions and manipulations (e.g. post-mortem stretching, in vitro chambers), to acquire distended or contracted bladders [7], [32], [33], we collected bladders just before and immediately after micturition in order to obtain physiologically distended and contracted bladder, respectively. During bladder filling, the shape of umbrella cells changed from cubical to flattened, the distinction between cells subapical and central cytoplasm became less obvious, but the morphology of mFVs did not change (Figs. 4A–G).

The reorientation of mFVs was observed in the subapical cytoplasm during distension and contraction of the urinary bladders. In the contracted bladders, 62% of mFVs were oriented perpendicular and 38% parallel to the apical cell surface (Fig. 4A). Contacts of hinge regions of mFVs with the apical plasma membrane were observed (Figs. 4B–F). In the distended bladder, 35% of mFVs were oriented perpendicular and 65% parallel to the apical cell surface (Fig. 4G). The difference was statistically significant (p = 0, 0375).

### Mature fusiform vesicles are limited to umbrella cells

**Figure 5:**
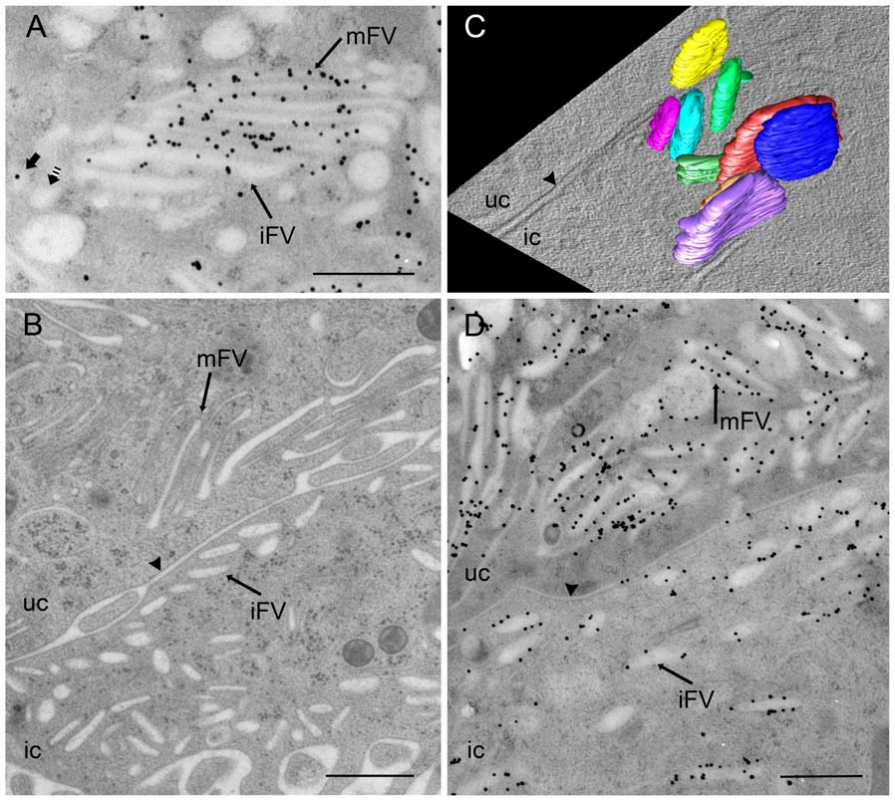
Mature FVs are only present in umbrella cells. (A) In umbrella cells, some stacks of FVs contain mFVs, which are densely labelled with anti-AUM antibody, and iFVs, which are shorter and more convex. Such stacks are surrounded by rounded uroplakin-positive (short black arrow) and uroplakin-negative (striped arrow) vesicles. (B) In the intermediate cell (ic), mFVs are not present; instead iFVs are seen below the plasma membrane (arrowhead) that borders the basolateral plasma membrane of the umbrella cell (uc). (C) Three-dimensional model of iFVs in the intermediate cell. (D) iFVs of intermediate cell (ic) are less densely labelled with anti-AUM antibody than mFVs of umbrella cell (uc). Bars: 500 nm.

Umbrella cells contained numerous vesicles, which mainly represented individually positioned or stacked mFVs described above. Occasionally, stacks were not uniform; they had mFVs only at one side of the stack, while on the other side of the stack vesicles were shorter and more convex. These types of the stack were surrounded by small round vesicles (Fig. 5A). The latter were either uroplakin-negative or uroplakin-positive. This observation pointed to the dynamics of membrane traffic between FVs and other cellular compartments during possible maturation process of FVs, which was recently described [18].

In the intermediate cells, typical mFVs were not found. Instead, uroplakin positive vesicles were smaller (up to 400 nm in length), and not as flat as mFVs of superficial umbrella cells (Fig. 5B). They contained smaller urothelial plaques compared to plaques of mFVs. These iFVs were concentrated beneath the plasma membrane of intermediate cells, which was in contact to the basolateral plasma membrane of umbrella cells. Three-dimensional reconstructions confirmed that iFVs were more convex than mFVs (Fig. 5C, Table S1 and Movies S5, S6) and they were also significantly less densely labelled with anti-AUM antibody than mFVs of umbrella cells (Fig. 5D).

## Discussion

FVs are highly specialized compartments that transport urothelial plaques in umbrella cells of the urothelium [20], [34]. Here we employed ET, freeze-fracturing and immuno-electron microscopy of the mouse urothelium in order to resolve the 3D ultrastructure and organization of FVs in their ‘close to native’ state. Our results indicate that rapid cryo-fixation is the superior method for ultrastructural studies of FVs and their morpho-functional organization in umbrella cells (Fig. 1).

Until now, conclusions about the nature of FVs were based on observations of ultrathin sections and freeze-fracturing whereas 3D models were not done yet. To investigate cellular structures in 3D rather than their 2D projections, ET is the method of choice [28]. However, FVs are relatively large compartments (up to 1.2 µm), and as such they cannot be included into a single semithin (200–300 nm) section. To make a full 3D model of mFVs, we therefore joined tomograms of multiple serial semithin sections. In that way we confirmed that mFVs are flattened discs (Fig. 2) as predicted earlier [6], [7]. They are composed of two opposing urothelial plaques that are connected along their rims by a flexible non-thickened membrane, which makes a distinct curvature [35].

The flattened disk shape of mFVs is in agreement with the function of urothelium. Secretory tissues contain numerous secretory vesicles, which are usually spheres with substantial intravesical volume filled with secretion products that are transported to the cell surface. Urothelium, on the other hand, does not secrete soluble products. Instead, the main products of urothelial umbrella cells are urothelial plaques, which function as blood-urine permeability barrier and help to accommodate the apical surface area of umbrella cells [5], [20]. Therefore, flattened disks with minimal intravesical lumen are perfectly shaped compartments to store and transport urothelial plaques to the apical plasma membrane on one hand, and to minimise internalization of toxic substances from urine on the other hand [36]. The mechanical stability of mFVs shape is probably provided by the rigidity of urothelial plaques [22], which prevents bending and ensures minimal lumen within mFVs.

To provide additional information on the higher organization of mFVs in urothelial umbrella cells, we made ET of large cell volumes. The results showed that mFVs are organized differently in the central and in the subapical cytoplasm of umbrella cells (Fig. 3). The separation of the central cytoplasm from the subapical cytoplasm is provided by a dense cytoskeletal network, formed by cytokeratins [30]. This network limits the exchange of FVs between the central and subapical cytoplasm of the cell. FVs may be transported from central to subapical cytoplasmic regions only through specialized openings in the cytokeratin network, called trajectories [24], [30]. This, and possibly the organization into stacks, may provide a mechanism, which limits and regulates the traffic of FVs between the central cytoplasm and the subapical cytoplasm.

In the central cytoplasm, FVs are often arranged into stacks of multiple FVs (Fig. 3). This may have two important functional consequences. First, when FVs are positioned close to each other, their maturation is facilitated. It has been suggested that FVs gradually mature by accumulation and expansion of crystalline arrays of 16-nm uroplakin particles, which derive from the *trans*-Golgi network [5], [6], [16], [18], [20], [37]. Second, the arrangement of mFVs into stacks, together with their flattened shape provides an ideal form for packing large areas of membranes into a small cell volume. The surface area of an average mFV (Fig. 3C; diameter 780 nm, thickness 27 nm) is 1,02 µm^2^, which is 3,8 times more than it would be in spherical vesicle (radius 145 nm, surface area 0,266 nm^2^) with the same volume (0,013 µm^3^). The difference is even more prominent when more mFVs are packed into a stack. Calculation shows that 10 mFVs (Fig. 3B) could be packed into approximately 10 times smaller volume that the spherical vesicles with the same surface. A similar organization can be observed in the outer segments of light-sensing cells in retina, where highly stacked membrane discs carry rhodopsin proteins [38]. These shows that mFVs are highly specialized and organized compartments, which provide a large storage pool of urothelial plaques needed to accommodate apical plasma membrane during distension-contraction cycles of the bladder [20], [39].

In the subapical cytoplasm, the orientation of mFVs depends on the distension/contraction state of urinary bladders (Fig. 4). The long axes of mFVs tend to be perpendicular to the luminal membrane in contracted bladders and parallel to it in dilated bladders. Bladders can accommodate to stretching by incorporating vesicles from a cytoplasmic pool into the apical plasma membrane and it has been assumed that hinge regions facilitate membrane fusions [4], [32], [39]. Our observation of contacts between mFVs and the apical plasma membrane supports this hypothesis. Therefore, reorientations of mFVs might facilitate the insertion of mFVs into the apical plasma membrane. However, the methods used in this study do not allow the conclusions whether all FVs in the subapical cytoplasm are destined to be inserted into the apical plasma membrane. It is possible that some FVs are derived from the apical surface. Our results of Rab27b immunolabelling support the idea of two classes of FVs, i.e. exocytotic and endocytotic [40]. However, the bladder state has no influence on the size and shape of the plaques.

Reorganization of mFVs from stacks in the central cytoplasm to individual entities in the subapical cytoplasm might reflect distinct roles of mFVs; stacked FVs enable maturation and storage of plaques, while individual mFVs facilitate the transport and fusion of plaques with the apical plasma membrane. Similar reorganizations of cellular compartments during protein transport was observed also in some other urothelium non-related cell systems reorganization [41].

Comparison with umbrella cells shows that intermediate cells contain vesicles that are smaller and less flattened as mFVs (Fig. 5). They are weakly labelled with antibodies against uroplakins; they contain smaller plaques and therefore they represent iFVs [18]. Interestingly, we could not detect fusions of iFVs with the plasma membrane of intermediate cells. A possible explanation for this is, that iFVs of intermediate cells lack a machinery for docking and/or fusion like specific Rab and SNARE complexes [42]. It can be stated that the maturation of FVs coincide with the terminal differentiation of urothelial cells. Smaller, more convex iFVs are therefore characteristic of partially differentiated intermediate cells, which contain less uroplakins and also lack cell surface-associated plaques [2], [10], [22], [43]. Large, flattened mFVs are typical compartments only of highly differentiated umbrella cells.

In conclusion, we have shown here 3D ultrastructure and higher organization of FVs in the urothelial cells. mFVs, present exclusively in terminally differentiated umbrella cells, are flattened discs that are organised into stacks in the central cytoplasm. From there, individual mFVs can be transported to the subapical cytoplasm, where their orientation greatly depends on the distension-contraction cycle of the urinary bladders. Due to their shape and higher organization, mFVs act as ideal compartments, which can store and transport large amount of membranes while occupying minimal volume of umbrella cells.

## Materials and Methods

### Animals

All animal experiments were approved by the Veterinary Administration of the Slovenian Ministry for Agriculture and Forestry (permission no. 34401-5/2009/4) and were in accordance with European guidelines and Slovenian legislation. Adult, male mice were used. They were bred under constant housing conditions, fed standard laboratory chow and water was available *ad libitum*. A regular circadian cycle was maintained for 12 hours; the experiments were conducted between 8 and 12 o'clock, which was the beginning of mice's night cycle. Contracted bladders were obtained immediately after micturition. In order to obtain distended bladders, the ligature was made on the penis after the micturition and animals were left in the cages for 3 hours before fixation.

### Chemical fixation

Mice were anaesthetized with ketamine/xylazine/atropine mixture (150 mg ketamine, 10 mg xylazine, and 0.1 mg atropine per kg body weight) and perfused with fixative through the left ventricle according to the method of Sprando [44]. For morphological study, 4% paraformaldehyde plus 2.5% glutaraldehyde in cacodylate buffer and for immunocytochemistry, 2% paraformaldehyde plus 0.05% glutaraldehyde in PBS were used. After fixation, abdominal cavities were opened, bladders removed and tissues were dissected in the same fixative as used for perfusion.

For morphological study, samples were put in the fixative for additional 2.5 hours, post fixed in 1% OsO_4_, dehydrated in graded ethanol and embedded in Epon. Ultrathin sections were counterstained with lead citrate and uranyl acetate.

For immunocytochemistry, samples were fixed for additional 1 hour, dehydrated and embedded in Lowicryl HM20 resin (PolysciencesInc, Warrington, USA) in Leica AFS apparatus (Leica Microsystems, Wetzlar, Germany) by the following schedule: dehydration in 30% ethanol for 30 min at 0°C, 55% ethanol for 30 min at −15°C, 70% ethanol for 30 min at −30°C, 100% ethanol for 60 min at −50°C, infiltration at −50°C in 75% ethanol/25% HM20 for 60 min, 50% ethanol/50% HM20 for 60 min, 25% ethanol/75% HM20 for 60 min, 100% HM20 for 60 min and 100% HM20 overnight. Blocks were polymerised under UV light for 48 hours at −50°C, then 3.5 hours at a 20°C/hour rising temperature and 24 hours at +20°C.

### High pressure freezing

Animals were killed with CO_2_, abdominal cavities were opened and exposed bladders were flushed from serosa side with cold PBS containing 4% paraformaldehyde for 10 seconds, to prevent muscular contraction during sectioning. Bladders were removed, rinsed quickly with PBS and transferred to 6-hexadecene. Tissue was cut into pieces with 2 mm diameter to fit freezing disks, and immediately frozen with liquid nitrogen at 2100 bar in a Balzers HPM 010 apparatus (Boeckeler Instruments, Tuscon, USA).

For morphological study and for ET, samples were freeze-substituted and embedded in Leica AFS (Leica Microsystems) apparatus according to Monaghan et al [45]: warming of samples to −90°C, freeze substitution in acetone containing 2% OsO_4_ at −90°C for 8 hours, at −60°C for 8 hours, and at −30°C for 8 hours. Substitution solution was changed with 100% acetone, warmed to 20°C and embedded in Epon.

For immunocytochemistry, frozen samples were warmed to −90°C and freeze-substituted in pure acetone at −90°C for 24, at −80°C for 120 and at −50°C for 12 hours. Acetone was replaced with 100% ethanol for 1 hour and samples were embedded in Lowicryl HM20 as described above for chemical fixation.

### Immunolabelling for light microscopy

Bladders were fixed with 4% paraformaldehyde in PBS for 1 hour, rinsed in PBS, embedded into 10% gelatine blocks, cryoprotected by incubation in cold 2.3 M saharose for 24 hours, and frozen in liquid nitrogen. Semithin cryo-sections (300 nm thick) were cut with Leica FCS cryo-ultramicrotom at −120°C and collected on coverslips. Nonspecific labelling was blocked as for TEM immunolabelling. Sections were incubated in cocktail containing anti-AUM antibody and mouse monoclonal anti-cytokeratin 20 antibody (Dako, Glostrup, Denmark) overnight at 4°C, washed in PBS, and then incubated with goat anti-rabbit and goat anti-mouse secondary antibodies, conjugated with AlexaFluor 555 and 488 (Invitrogen, Carlsbad, USA), respectively, at room temperature for 1.5 hours. Negative controls were done by omitting the primary antibodies, by incubation in rabbit serum, or by using inadequate primary antibodies. Sections were examined with a T300 (Nikon, Tokyo, Japan) fluorescence microscope.

### Immunolabelling for TEM

#### Lowicrly embedded sections

Lowicryl embedded ultrathin sections from chemically fixed and high pressure frozen samples were processed according to the same protocol. Non-specific labelling was blocked by the PBS buffer containing 0.1% fish gelatine, 0.8% bovine serum albumin and 5% fetal calf serum (blocking buffer). Sections were incubated with polyclonal antibody made against highly purified bovine asymmetric unit membrane (anti-AUM antibody, a kind gift of prof. TT Sun, NYU), which reacts strongly with uroplakin IIIa, moderately with uroplakin Ia and Ib, and weakly with uroplakin II [10]. After rinse in washing buffer (blocking buffer without fetal calf serum), anti-AUM was detected with goat anti-rabbit IgG conjugated to 5 nm colloidal gold diluted 1∶50 in blocking buffer. Sections were washed in washing buffer followed by de-ionized water. The 5 nm gold was silver enhanced with IntenSE (GE Healthcare, Amersham, UK). The enhancement times were 4 minutes. Negative controls were done by omitting the primary antibodies, by incubation in rabbit serum, or by using inadequate primary antibodies.

#### Ultrathin cryo-sections

Ultrathin cryo-sections were prepared by modified Tokuyashu method. Briefly, urothelium, cut into <1 mm^3^ pieces, was fixed in 4% paraformaldehyde plus 0.1% glutaraldehyde 0.1 M phosphate buffer for 2 hours, washed in PBS, embedded in 12% gelatine and cryoprotected by incubation in 2.3 M saharose. Samples were then stored in liquid nitrogen, and were subsequently cut with a Leica FCS cryo-ultramicrotom at −120°C, and processed for immunolabelling with anti-Rab27b rabbit polyclonal antibody (SigmaAldrich, St. Louis, USA). Primary antibodies (diluted 1∶10000) were incubated overnight at 4°C, washed in PBS, and incubated with goat anti rabbit secondary antibodies, conjugated with 10 nm colloidal gold (SigmaAldrich, diluted 1∶40), at room temperature for 1.5 hours. Blocking non-specific labelling and negative controls were done as for Lowicryl embedded ultrathin sections.

All immunolabelled sections were viewed in a Philips CM100 (Eindhoven, Netherlands) electron microscope, running at 80 kV.

### Freeze-fracturing

Urothelium was fixed in 2.5% glutaraldehyde and 4% paraformaldehyde in 0.1 M cacodylate buffer, pH 7.3, for 2 hours at 4°C. Tissue pieces were soaked in 30% glycerol in 0.1 M cacodylate buffer for 2 hours before freezing in liquid nitrogen. Replicas were produced in a Balzers BAF 301 freeze-fracture unit. Replica were cleaned by the technique of Van den Bosch and Jacob [46] and examined in the electron microscope.

### Electron tomography

Semithin sections (200–300 nm thick) from high pressure frozen, Epon embedded tissues were cut perpendicular to the apical surface of the urothelium. ET was performed in a Tecnai-20 transmission electron microscope (FEI, Eindhoven, Netherlands) equipped with a tilting stage and running at 200 kV. A rotation holder (Gatan, Inc., Pleasanton, USA) was used in order to orient the sections. Series of tilted images (range: −65° to +65°) were acquired with a tilt increment of 1° by the help of the Xplore 3D software (FEI). This software requires a holder calibration procedure where the dislocation and the defocus of every tilt image are recorded. Before acquisition of a tilt series, an automatic adjustment of the eucentric height and an autofocus procedure were performed. During acquisition of tilted images dislocations caused by the mechanical imprecision of the stage are corrected. The volume of the semithin sections was reconstructed into serial slices using the software package Inspect 3D (FEI). This software implicates alignment and filter tools as well as a reconstruction module involving Fourier transforms and weighted back projection of the 2D tilt images.

For studying whole 3D morphology and higher organization of FVs, ten serial semithin sections were cut and all sections were examined in order to find recognisable FVs that could be followed throughout their whole volume. For high resolution reconstructions of individual FVs and of their higher organization, ET was done at magnifications 5000–9600×. Modelling was done using IMOD software (http://bio3d.colorado.edu/imod/) [47]. Tomograms from each of the serial sections were joined along the Z-axis as described previously [48]. Supplement movies of the FVs models were compiled using MovieMaker 2.6 software (Microsoft, Redmond, USA) from sequences of individual JPEG files generated using the 3dmod viewer in IMOD.

### Quantitative analysis

For quantitative analysis of mFVs orientation relative to the apical plasma membrane of umbrella cells, 5 contracted and 5 distended urinary bladders were used. Ultrathin sections (60 nm thick) were prepared from two Epon blocks of each animal and micrographs of 10 umbrella cells were taken at a magnification 21000× in a Philips CM100 microscope, running at 80 kv. Only umbrella cells with folded or flattened apical plasma membrane for contracted or distended urothelium, respectively, were analysed. In these cells, orientation and number of mFVs lying less than 1000 nm below the apical plasma membrane was determined. Orientation of FVs was determined as the angle of mFVs long-axis relative to the apical plasma membrane. Measurements were done on a personal computer using ImageJ ver. 1.44 software for Windows [49]. FVs, with their relative angles less than 45° were labelled as FVs parallel to the apical plasma membrane, while FVs, with their relative angles more than 45° were labelled as FVs perpendicular to the apical plasma membrane. Statistical analyses were performed with Microsoft Office Excel 2007 software. Statistical significance was tested with Student's t-test.

## Supporting Information

Figure S1 **Position of cytokeratin 20 network in the urothelial cells.** Immunolabelling with anti-CK20 (green) antibody shows that cytokeratin 20 is distributed as a line (arrow) below the luminal membrane of umbrella cells (uc). Majority of FVs, immunolabelled with anti-AUM antibody (red), are positioned in the central cytoplasm beneath the cytokeratin 20 location. Legend: blue – DAPI, ic - intermediate cell, bc – basal cell. Thickness of semithin cryo-section is 300 nm. Bar: 5 µm.
(TIF)

Movie S1 **Tomogram of a single mature FV in the umbrella cell.**
(WMV)

Movie S2 **Single mature FV in the umbrella cell.**
(WMV)

Movie S3 **Tomogram of stack of mature FVs in the central cytoplasm of umbrella cell.**
(WMV)

Movie S4 **Stack of mature FVs in the central cytoplasm of umbrella cell.**
(WMV)

Movie S5 **Tomogram of immature FVs in the intermediate urothelial cell.**
(WMV)

Movie S6 **Immature FVs in the intermediate urothelial cell.**
(WMV)

Table S1 **Data of the tomograms shown in** **Figures 2** **,** **3** **and** **5** **.**
(DOC)
