# Supplementary figures and images for: Electron Tomography of Fusiform Vesicles and Their Organization in Urothelial Cells
Source: PLoS One. 2012 Mar 12;7(3):e32935. doi: 10.1371/journal.pone.0032935 (PMC3299716; doi:10.1371/journal.pone.0032935)

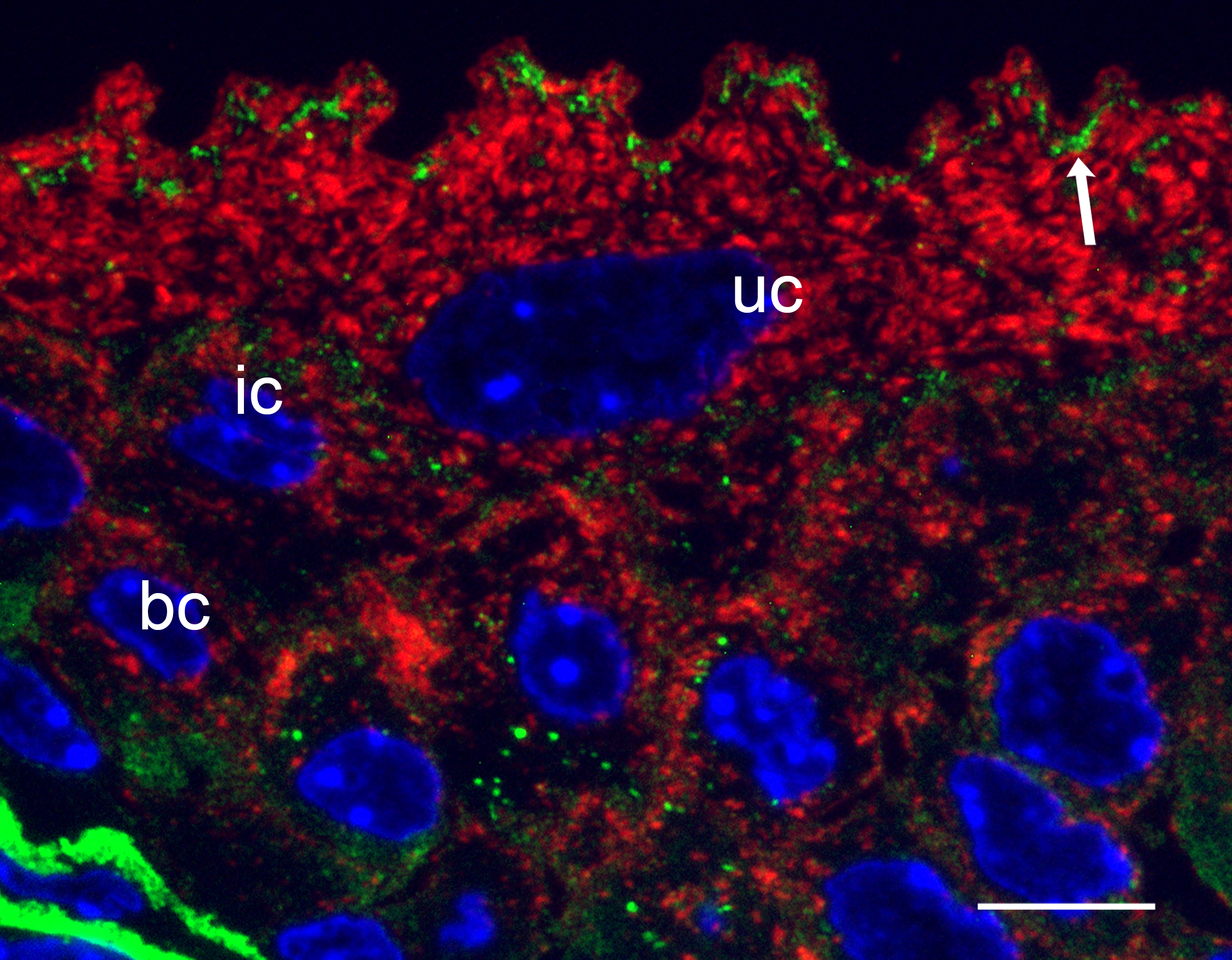

Supplement: Figure S1 — Position of cytokeratin 20 network in the urothelial cells. Immunolabelling with anti-CK20 (green) antibody shows that cytokeratin 20 is distributed as a line (arrow) below the luminal membrane of umbrella cells (uc). Majority of FVs, immunolabelled with anti-AUM antibody (red), are positioned in the central cytoplasm beneath the cytokeratin 20 location. Legend: blue – DAPI, ic - intermediate cell, bc – basal cell. Thickness of semithin cryo-section is 300 nm. Bar: 5 µm. (TIF) [file pone.0032935.s001.tif]
