# Supplementary material for: Electron Tomography of Fusiform Vesicles and Their Organization in Urothelial Cells
Source: PLoS One. 2012 Mar 12;7(3):e32935. doi: 10.1371/journal.pone.0032935 (PMC3299716; doi:10.1371/journal.pone.0032935)
Supplement: Table S1 — Data of the tomograms shown in Figures 2 , 3 and 5 . (DOC) [file pone.0032935.s008.doc]

**Table S1: Data of the tomograms shown in Figures 2, 3 and 5.**

| Tomogram of the model shown on | Figs. 2D-G | Fig. 3D | Fig. 5C |
| --- | --- | --- | --- |
| Magnification | 5000 x | 7800 x | 9600 x |
| Start/stop tilt; step | -60º /+65º; 1º | -60º /+65º; 1º | -55º /+55º; 1º |
| Pixel size | 3.48 nm | 2.27 nm | 1.83 nm |
| REC sections | 770 | 201 | 281 |
